# Alterations in the mammary gland and tumor microenvironment of formerly obese mice

**DOI:** 10.1186/s12885-023-11688-3

**Published:** 2023-12-01

**Authors:** Genevra Kuziel, Brittney N. Moore, Grace P. Haugstad, Yue Xiong, Abbey E. Williams, Lisa M. Arendt

**Affiliations:** 1 Cancer Biology Program, University of Wisconsin-Madison, Madison, WI 53705 USA; 2 Department of Comparative Biosciences, University of Wisconsin-Madison, Madison, WI 53706 USA; 3 Comparative Biomedical Sciences Program, University of Wisconsin-Madison, Madison, WI 53706 USA; 4 School of Veterinary Medicine, 2015 Linden Drive Rm 4354A, Madison, WI 53706 USA

**Keywords:** Mammary gland, Obesity, Fibrosis, Fibrocytes, Macrophages, Weight loss, Breast cancer, Myeloid progenitor cells, Collagen

## Abstract

**Background:**

Obesity is a risk factor for breast cancer, and women with obesity that develop breast cancer have a worsened prognosis. Within the mammary gland, obesity causes chronic, macrophage-driven inflammation and adipose tissue fibrosis. Weight loss is a recommended intervention to resolve obesity, but the impact of weight loss on the mammary gland microenvironment and in tumors has not been well identified.

**Methods:**

To examine the effects of weight loss following obesity, mice were fed a high-fat diet for 16 weeks to induce obesity, then switched to a low-fat diet for 6 weeks. We examined changes in immune cells, including fibrocytes, which are myeloid lineage cells that have attributes of both macrophages and myofibroblasts, and collagen deposition within the mammary glands of non-tumor-bearing mice and within the tumors of mice that were transplanted with estrogen receptor alpha positive TC2 tumor cells.

**Results:**

In formerly obese mice, we observed reduced numbers of crown-like structures and fibrocytes in mammary glands, while collagen deposition was not resolved with weight loss. Following transplant of TC2 tumor cells into the mammary glands of lean, obese, and formerly obese mice, diminished collagen deposition and cancer-associated fibroblasts were observed in tumors from formerly obese mice compared to obese mice. Within tumors of obese mice, increased myeloid-derived suppressor cells and diminished CD8^+^ T cells were identified, while the microenvironment of tumors of formerly obese mice were more similar to tumors from lean mice. When TC2 tumor cells were mixed with CD11b^+^CD34^+^ myeloid progenitor cells, which are the cells of origin for fibrocytes, and transplanted into mammary glands of lean and obese mice, collagen deposition within the tumors of both lean and obese was significantly greater than when tumor cells were mixed with CD11b^+^CD34^−^ monocytes or total CD45^+^ immune cells.

**Conclusions:**

Overall, these studies demonstrate that weight loss resolved some of the microenvironmental conditions within the mammary gland that may contribute to tumor progression. Additionally, fibrocytes may contribute to early collagen deposition in mammary tumors of obese mice leading to the growth of desmoplastic tumors.

**Supplementary Information:**

The online version contains supplementary material available at 10.1186/s12885-023-11688-3.

## Background

Global obesity rates are continuing to rise [1, 2]. Obesity significantly increases the risk for the development of hormone receptor positive breast cancer in postmenopausal individuals [3–5]. Breast cancer patients with obesity have a significantly worse prognosis and overall survival regardless of menopausal status or tumor subtype [6]. Further, breast tumors from patients with obesity demonstrated higher levels of desmoplasia, which is characterized by increased alpha-smooth muscle actin (SMA) positive cancer-associated fibroblasts (CAF) and collagen deposition, than breast tumors from lean patients [7], suggesting that obesity also impacts the breast tumor microenvironment.

Weight loss ameliorates multiple health conditions associated with obesity, and epidemiological studies have shown that weight loss may decrease the risk for breast cancer in women with obesity [8, 9]. Breast tissue is a depot of subcutaneous adipose tissue, and a hallmark of obesity is the recruitment of macrophages to form crown-like structures (CLS) to remove lipid and necrotic adipocytes [10]. In patients treated with bariatric surgery for weight loss, macrophage populations appear to switch from an inflammatory to an alternatively activated phenotype in subcutaneous white adipose tissue [11, 12], which may enhance tissue repair [13]. The impact of weight loss on adipose tissue fibrosis is less clear [14, 15]. Changes in inflammation and adipose tissue fibrosis following weight loss have not been investigated in the mammary gland. Further, limited mouse models have examined how weight loss affects tumor growth and the tumor microenvironment. The effects of weight loss prior to tumor formation on the resulting mammary tumor microenvironment have yet to be examined.

In obesity, mammary adipose tissue is associated with increased collagen deposition and stiffness surrounding adipocytes [7, 16] and the emergence of SMA^+^ myofibroblasts [7]. We have shown that fibrocytes are increased in obesity and contribute to fibrosis in the mammary gland [17]. Fibrocytes, which originate in the myeloid progenitor cell population of the bone marrow, have attributes of both macrophages and myofibroblasts and are associated with diseases characterized by inflammation and fibrosis [18, 19]. Fibrocytes have been identified in tissues using combinations of markers including CD34, CD11b, CD45, SMA, and collagen I [20, 21]. Recent single cell RNA sequencing studies have identified a role for fibrocytes in the pathogenesis of lung tumors [22, 23]. In a tumor model of inflammation associated with obesity, we identified elevated numbers of fibrocytes in early-stage mammary tumors [24]. In human breast tissue, CD34^+^ cells have been detected in the extracellular matrix surrounding breast lobules and low grade ductal carcinoma in situ (DCIS) but not when SMA^+^ myofibroblasts were increased surrounding high grade DCIS and invasive ductal carcinoma [25, 26], which is suggestive of differentiating fibrocytes. However, the role of fibrocytes in altering the breast tumor microenvironment in obesity has not been examined.

Here, we investigate how weight loss impacts mammary gland inflammation and collagen fibrosis, as well as tumor growth and development of the tumor microenvironment, using a diet-induced obesity mouse model. We observed that weight loss resolves CLS and reduces fibrocytes within the mammary gland but does not change total numbers of macrophages or collagen deposition. Tumors that develop in the mammary glands of formerly obese mice have a tumor microenvironment more similar to tumors from lean mice. Interestingly, fibrocytes were decreased in tumors from obese mice. However, transplant of estrogen receptor alpha (ERα)^+^ TC2 tumor cells mixed with myeloid progenitor cells from the bone marrow of obese mice into the mammary glands of both lean and obese mice leads to lasting increases in collagen and CAF within tumors. Together, these results suggest that weight loss prior to tumor formation reduces desmoplasia within tumors, potentially through reduced numbers of fibrocytes.

## Methods

### Transgenic mice

All animal procedures were conducted under approved animal protocol V001588 in compliance with the guidelines and regulations of the University of Wisconsin Institutional Animal Care and Use Committee and housed in AAALAC accredited facilities (Animal Welfare Assurance Number: D16-00239). All methods are reported in accordance with ARRIVE guidelines for reporting of animal experiments. FVB.Cg-Tg(CAG-EGFP)B5Nagy/J mice (EGFP; 003516) [27] were purchased from Jackson Laboratory (Bar Harbor, ME, USA). FVB/NTac female mice were purchased from Taconic Biosciences. Mice were given food and water ad libitum and weighed weekly. Three-week-old female mice were randomized to receive either a low-fat diet (LFD; 16% kcal from fat; 2920X; Teklad Global; ENVIGO) or a high-fat diet (HFD; 60% kcal from fat; Test Diet, 58Y1) for 16 weeks. After 16 weeks on the HFD, mice were randomly selected to be switched to the LFD for 6 weeks to induce weight loss (WL). Mice were humanely euthanized using CO_2_ asphyxiation followed by cervical dislocation.

### Isolation of cells

To isolate immune cells, bone marrow was flushed from the humerus and femurs of tumor-bearing and non-tumor-bearing mice of all groups. Mammary glands and tumors were mechanically minced then enzymatically dissociated for 1 h (mammary gland) or 1.5 h (tumor) at 37 °C in DMEM (10–017-CV; Corning Inc., Corning, NY, USA) supplemented with 10% FBS, 1% antibiotic/antimycotic solution (30–004-CI; Corning, Inc.), 1.5 mg/mL collagenase A (11,088,793,001; MilliporeSigma, Burlington, MA, USA), and 125 U/mL hyaluronidase (H3506; Sigma-Aldrich, St. Louis, MO, USA). Both bone marrow and mammary glands were treated with ACK Lysing Buffer (10-548E; Lonza, Basel, Switzerland) to lyse red blood cells. Mammary glands and tumors were dissociated to single cells as described [28].

### Cell lines

Parental and GFP^+^ TC2 cells were obtained from the lab of Dr. Linda Schuler [29]. TC2 tumor cells were tested for mycoplasma using the Impact III Panel (Idexx Bioresearch). Parental TC2 cells were cultured in DMEM (10–017-CV; Corning Inc.) supplemented with 10% fetal bovine serum and 1% antibiotic/antimycotic solution (30–004-CI; Corning, Inc). GFP^+^ TC2 cells were cultured in complete media supplemented with 1 mg/ml Geneticin (G418 Sulfate) (11,811,023; ThermoFisher, Waltham, MA, USA). TC2 cells were validated for ERα and GFP expression prior to use in studies. Cells were cultured at 37 °C with 5% CO_2_.

### Flow cytometry and FACS isolation

Flow cytometry was performed and analyzed according to published guidelines [30]. Both bone marrow and mammary gland cells were prepared as previously described [24], with antibodies in Additional File 1. Flow cytometry was performed using a BD LSRFortessa (BD Biosciences; San Jose, CA, USA). Fluorescence-activated cell sorting (FACS) was performed using a BD FACS Aria III cell sorter (BD Biosciences; San Jose, CA, USA) at the Flow Cytometry Laboratory (Carbone Cancer Center, University of Wisconsin-Madison). Gates were set using fluorescence-minus-one (FMO) controls. Data were analyzed using FlowJo 10.8.1 (Becton, Dickinson and Company, Ashland, OR, USA).

### TC2 Mammary tumor transplant

Fifty thousand GFP^+^ TC2 tumor cells were mixed with 1:1 DMEM: Matrigel (354,234; Corning, Inc.) and injected into bilateral inguinal mammary glands of lean, obese, and formerly obese mice. Two experimental cohorts were combined for each group. For transplant of TC2 tumor cells mixed with bone marrow cells, FACS was used to isolate total CD45^+^ cells, CD45^+^CD11b^+^CD34^−^ monocytes, and CD45^+^CD11b^+^CD34^+^ myeloid progenitor cells from bone marrow of obese EGFP mice. 25,000 FACS-isolated bone marrow cells were mixed with 50,000 parental TC2 cells and injected into mammary glands of lean and obese mice. Tumor length and width were recorded once a week with calipers. Tumor volume was calculated using the formula (L*W*W)/2. Mice were humanely euthanized and tumors collected, once tumors reached 1 cm in length for tumor growth in lean, obese, or formerly obese mice or 0.7 cm in length in transplantation of bone marrow studies.

### Immunomagnetic cell sorting and fibrocyte culture

Immunomagnetic bead sorting was performed on mammary glands and tumors as previously described [24], with the following modifications. CD11b^+^ cells were plated at 20,000 cells/well on 12-well plates. Following blinding, colonies were counted 5 days after plating. Fibrocyte colonies grown for immunofluorescent staining were plated on 8-well chamber slides (15,434; ThermoFisher) coated with poly-L-lysine (PLL; P4707; Sigma-Aldrich) and grown for 10 days. All sorted cells were grown in Mouse MesenCult Expansion Kit Media (05513; StemCell Technologies, Vancouver, BC, Canada). Cells were cultured at 37 °C with 5% CO_2_. To image colonies, cells were fixed with cold 100% methanol for 20 min at -20 °C and then stained with 0.1% crystal violet. Images were captured using a Keyence BZ-X710 microscope (Itasca, IL).

### Histology and immunofluorescence

Tissue was fixed in 10% neutral buffered formalin for 48 h, then paraffin-embedded and sectioned by the Experimental Animal Pathology Laboratory (Carbone Cancer Center, University of Wisconsin-Madison). Picrosirius red staining to detect collagen was completed as described [31]. Immunohistochemistry and immunofluorescence were performed as described [32]. All antibodies are listed in Additional File 1. Slides stained for quantification were blinded, then imaged with identical image acquisition settings using a Leica TCS SP8 Confocal Microscope (Leica Microsystems, Buffalo Grove, IL, USA) or a Keyence BZ-X710 microscope. For CLS quantification, five random 100 × images were taken of the mammary fat, and CLS were quantified and averaged for each mouse. Picrosirius red staining around mammary ducts and tumors was imaged and quantified as described [32]. Briefly, picrosirius red staining around mammary ducts was imaged along with tissue autofluorescence and quantified using ImageJ. For each image, the autofluorescence was subtracted from the picrosirius red staining. The image was then made binary, and collagen pixels were quantified by tracing. By inverting the image, the area of the duct was also quantified by tracing, and a ratio was calculated for the ratio of collagen:duct area. Five mammary ducts were quantified per mammary gland, and the values were averaged. Picrosirius red and SMA stained tumor sections were tile-scanned in total and stitched using BZ-X Analyzer Software 1.3.0.3 (Keyence). SMA staining was quantified using ImageJ. A ratio was calculated for SMA area: total area. F4/80 staining within tumors was quantified using the Color Deconvolution 2 ImageJ plugin [33].

### Statistical analysis

Significance was determined at *p*-values of 0.05 or less. Data were tested for normality using the Shapiro–Wilk test prior to further statistical analysis. One-way ANOVA with Tukey’s multiple comparisons test was used unless stated for data with a normal distribution. For data with a distribution that was not normal, we used Kruskal–Wallis with Dunn’s multiple comparison post-test. Two-way ANOVA with Tukey’s multiple comparison post-test was used to test changes in mouse weight over time. To test differences in weight following weight loss, a paired t-test was used. Outliers were detected using Grubbs’ test. Correlation was determined using Spearman correlation, and best fit line generated using linear regression. Sample sizes were calculated for weight loss and tumor growth studies with a power of 0.8. Error bars represent mean ± S.E.M. unless stated. Statistical analyses were conducted using GraphPad Prism 9.4.1 (GraphPad Software, San Diego, CA, USA).

## Results

### Weight loss partially resolves inflammation but not fibrosis

**Fig. 1:**
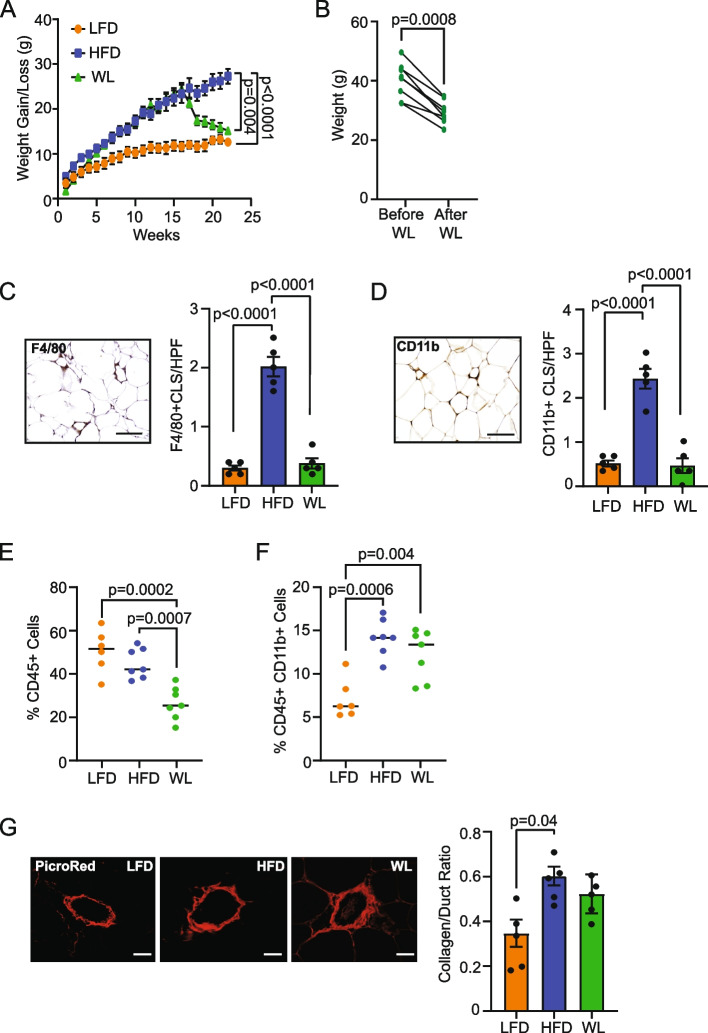
Weight loss partially resolves characteristics of obesity in mammary glands. **A** 3-week-old female FVB/N mice were fed low-fat diet (LFD) or high-fat diet (HFD) for 16 weeks, then a cohort of the HFD group was switched to LFD for 6 weeks for weight loss (WL; *n* = 8–10/group; Two-way ANOVA with Tukey’s multiple comparison test). **B** Weight change of mice switched from HFD to LFD for 6 weeks (paired t-test). **C** Representative image of F4/80^+^ CLS per high power field (HPF) and quantification. **D** Representative image of CD11b^+^ CLS per HPF and quantification. **E** The percentage of live CD45^+^ cells in mammary glands of lean, obese, and formerly obese mice quantified by flow cytometry. **F** The percentage of live cells positive for both CD45^+^ and CD11b^+^ in mammary glands quantified by flow cytometry. **G** The ratio of the area picrosirius red-stained collagen surrounding mammary ducts to duct area. Magnification bar: (**C, D**) 50 µm; (**G**) 25 µm

We have previously observed that mice switched from HFD to LFD lose a significant amount of weight and resolve obesity-induced changes in mammary epithelial cell populations [28]. To investigate the effects of weight loss on stromal cells in the mammary gland, female FVB/N mice were fed either LFD or HFD for 16 weeks, then a group of mice fed HFD was switched to the LFD to induce weight loss (Fig. 1A). These mice rapidly lost a significant amount of weight (*p* < 0.0008; Fig. 1B). Within the mammary glands of formerly obese mice, we observed significantly reduced numbers of CLS formed by both F4/80^+^ macrophages (*p* < 0.0001; Fig. 1C, Additional File 2A) and CD11b^+^ myeloid lineage cells (*p* < 0.0001; Fig. 1D, Additional File 2B) compared to HFD-fed mice. These results demonstrate a loss of this signature of adipose tissue inflammation with weight loss.

To further examine how weight loss impacts myeloid lineage cells, mammary glands from lean, obese, and formerly obese mice were dissociated to single cells and stained with antibodies to detect CD45, CD11b, and CD34 by flow cytometry (Additional File 2C). The percentage of total live cells that were positive for CD45 was decreased in formerly obese mice compared to either lean (*p* = 0.0002) or obese mice (*p* = 0.007; Fig. 1E). However, the percentage of live cells that expressed both CD45 and CD11b, indicative of myeloid cells, was significantly increased in both obese (*p* = 0.0006) and formerly obese mice (*p* = 0.004) compared to lean mice (Fig. 1F). These data indicate that obesity induces an influx of myeloid cells into the mammary gland, which is not resolved after 6 weeks of weight loss.

Obesity has been shown to increase fibrosis in the mammary gland [34, 35]. To determine if fibrosis is reduced with weight loss, collagen surrounding ducts was quantified using picrosirius red staining in mammary glands of lean, obese, and formerly obese mice (Additional File 2D). Duct area was not significantly different among the ducts that we imaged in any of the groups of mice (Additional File 2E), while collagen area was significantly increased surrounding the ducts in obese mice (*p* = 0.01, Additional File 2F). To adjust for any potential differences in duct size, we examined the ratio of the collagen to duct area. Obese mice had a significantly larger collagen:duct ratio compared to lean mice (*p =* 0.04, Fig. 1G), while the collagen:duct ratio in formerly obese mice was not significantly different from either obese or lean mice (Fig. 1G). These results show that while weight loss resolved CLS, fibrosis was a sustained part of the microenvironment.

### Weight loss decreases myeloid progenitor cells and fibrocytes

**Fig. 2:**
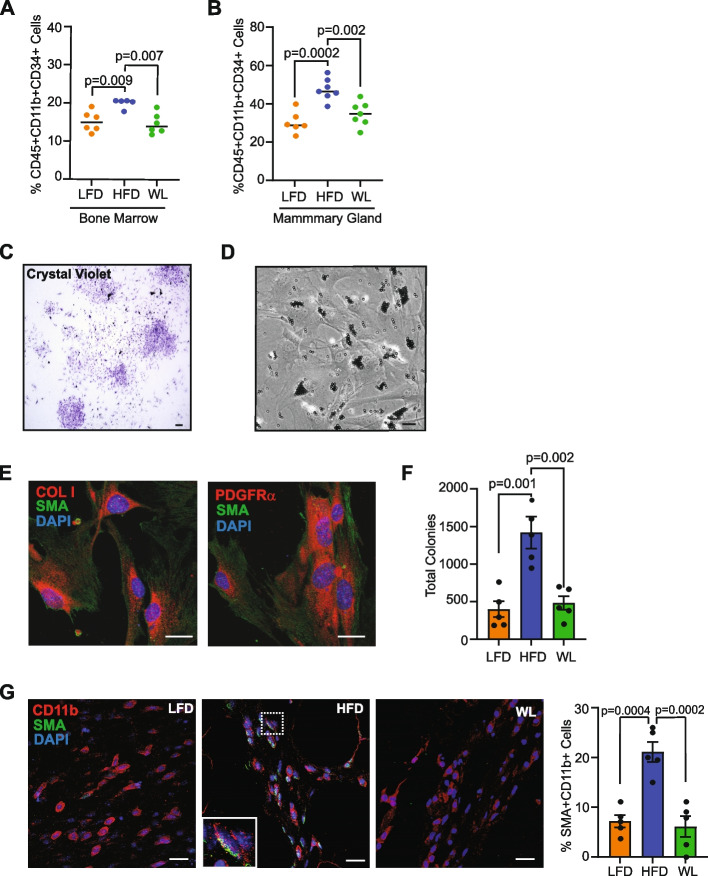
Weight loss reduces fibrocyte colony formation within the mammary glands. **A** The percentage of live CD45^+^CD11b^+^CD34^+^ myeloid progenitor cells in bone marrow quantified by flow cytometry. **B** The percentage of CD45^+^CD11b^+^CD34^+^ immature myeloid cells in mammary glands quantified by flow cytometry. **C** Representative crystal violet image of colonies grown from immunomagnetically sorted CD11b^+^ cells. **D** Representative phase contrast image of an adherent colony grown from CD11b^+^ cells. **E** Representative images of colonies grown from CD11b^+^ cells stained to detect SMA and collagen I and SMA and platelet-derived growth factor receptor alpha (PDGFRα). **F** Colony quantification of CD11b^+^ sorted cells from mammary glands. **G** Representative images of mammary glands stained for CD11b and SMA. Quantification of the percentage of CD11b^+^SMA^+^ cells/total CD11b^+^ cells. Magnification bar: (**C, D**) 100 µm; (**E, G**) 25 µm

Fibrocytes have been associated with fibrosis in multiple contexts and are thought to originate in the myeloid progenitor cell population of the bone marrow [18]. To assess the effects of weight loss on myeloid progenitor cells, we quantified bone marrow cells from lean, obese, and formerly obese mice using flow cytometry (Additional File 3A). The percentage of live cells that expressed CD45, CD11b, and CD34, indicative of myeloid progenitor cells, was increased in bone marrow of obese mice compared to lean mice (*p* = 0.009, Fig. 2A) and decreased in formerly obese mice compared to obese mice (*p *= 0.007, Fig. 2A). We have previously shown that fibrocytes arise from CD11b^+^CD34^+^ cells in the mammary gland [24]. We observed that the percentage of CD45^+^CD11b^+^CD34^+^ immature myeloid cells was significantly increased in the mammary glands of obese mice compared to lean mice (*p* = 0.0002) and reduced in formerly obese mice compared to obese mice (*p =* 0.002, Fig. 2B).

To examine the impact of weight loss on fibrocytes, CD11b^+^ cells were immunomagnetically sorted from the mammary glands of lean, obese, and formerly obese mice, and assessed for their ability to form adherent colonies in vitro (Fig. 2C). Fibrocytes formed adherent colonies that had a morphology similar to myofibroblasts (Fig. 2D) and expressed markers SMA, collagen I, and platelet-derived growth factor receptor alpha (PDGFRα) (Fig. 2E). Consistent with previous studies [17], expression of immune cell markers CD45 and F4/80 were not detectable within the colonies (Additional File 3B, C). CD11b^+^ cells isolated from obese mice generated significantly more fibrocyte colonies than lean mice (*p* = 0.001), while fibrocyte colony formation was significantly reduced in CD11b^+^ cells from formerly obese mice compared to those from obese mice (*p* = 0.002, Fig. 2F).

To examine fibrocytes within tissue, mammary glands from lean, obese, and formerly obese mice were stained for CD11b and SMA (Fig. 2G, Additional File 3D). CD11b^+^SMA^+^ cells were observed significantly more frequently in the collagen surrounding mammary ducts in the mammary glands of obese mice compared to either lean (*p* = 0.0004) or formerly obese mice (*p =* 0.0002, Fig. 2G). Altogether, these results suggest that weight loss reduces fibrocyte numbers within mammary glands.

### Weight loss prior to tumor growth reduces tumor fibrosis

**Fig. 3:**
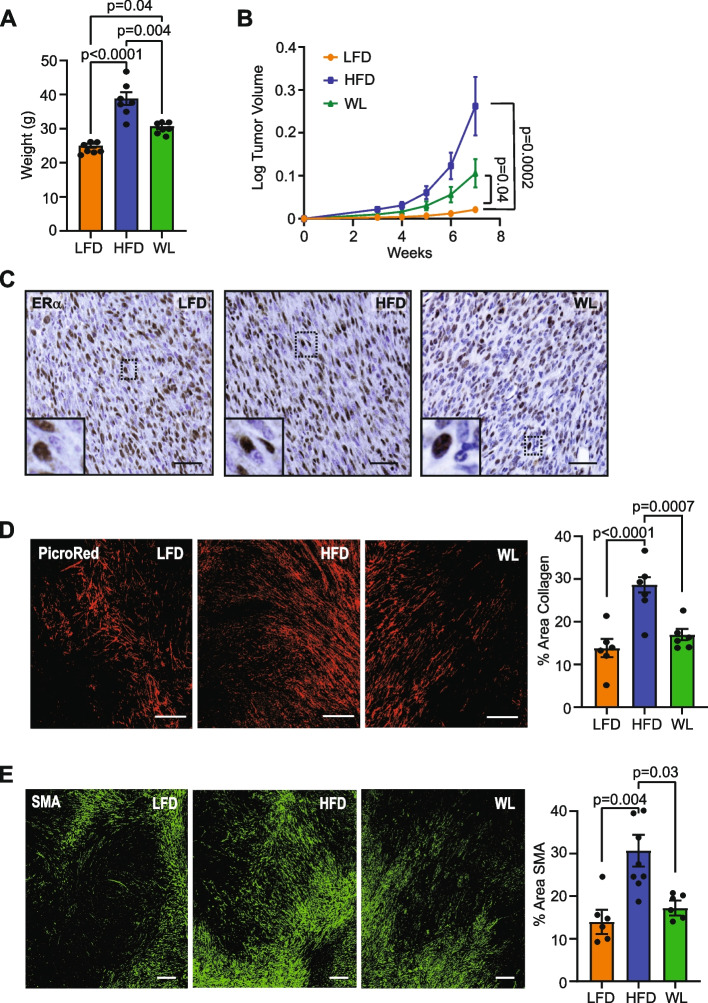
Weight loss preceding TC2 tumor cell transplant reduces CAF and collagen within tumor microenvironment. **A** Mouse weights at time of surgery. **B** TC2 tumor growth following injection of LFD, HFD, and WL inguinal mammary glands (Kruskal–Wallis with Dunn’s multiple comparisons test). **C** Representative images of ERα expression in TC2 mammary tumors. **D** Representative images and quantification of picrosirius red-stained collagen within tumors normalized to total tumor area. **E** Representative images and quantification of SMA expression within tumors normalized to total tumor area. Magnification bar: (**C**) 50 µm; (**D, E**) 300 µm

We have previously observed that obesity increases mammary tumor growth rates [36, 37]. To investigate the impact of weight loss on mammary tumor growth and the tumor microenvironment, we generated lean, obese, and formerly obese mice. Formerly obese mice weighed significantly less than obese mice (*p* = 0.004), but significantly more than lean mice (*p* = 0.04, Fig. 3A) at the time of tumor cell injection. ERα^+^ TC2 mammary tumor cells were injected into the mammary glands of mice in each group. Similar to our previous studies examining ERα^−^ tumor growth [36, 37], mammary tumors grew significantly faster in obese mice compared to lean mice (*p* = 0.0002, Fig. 3B). Mammary tumors in formerly obese mice grew significantly faster than tumors in lean mice (*p =* 0.04) and were not significantly different from tumors from obese mice (Fig. 3B). Mammary tumors retained ERα expression at end stage (Fig. 3C), and we have previously observed that ERα expression within tumors was not different in tumors of obese and lean mice [37].

To assess how weight loss prior to tumor growth impacted the mammary tumor microenvironment, tumors from lean, obese, and formerly obese mice were stained with picrosirius red and collagen deposition was quantified. Collagen was significantly increased within tumors of obese mice compared to lean mice (*p* < 0.0001, Fig. 3D), and collagen within tumors of formerly obese mice was significantly diminished compared to obese mice (*p* = 0.0007, Fig. 3D). Tumors were also stained with SMA to identify CAF. Obesity significantly increased CAF in tumors compared to lean mice (*p =* 0.004, Fig. 3E), while CAF were significantly reduced in tumors from formerly obese mice compared to obese mice (*p* = 0.03, Fig. 3E). Taken together, these data indicate that the mammary microenvironment after weight loss may no longer promote collagen deposition and CAF formation in mammary tumors while still contributing to tumor growth.

### Weight loss prior to tumor growth reduces an immunosuppressive microenvironment

**Fig. 4:**
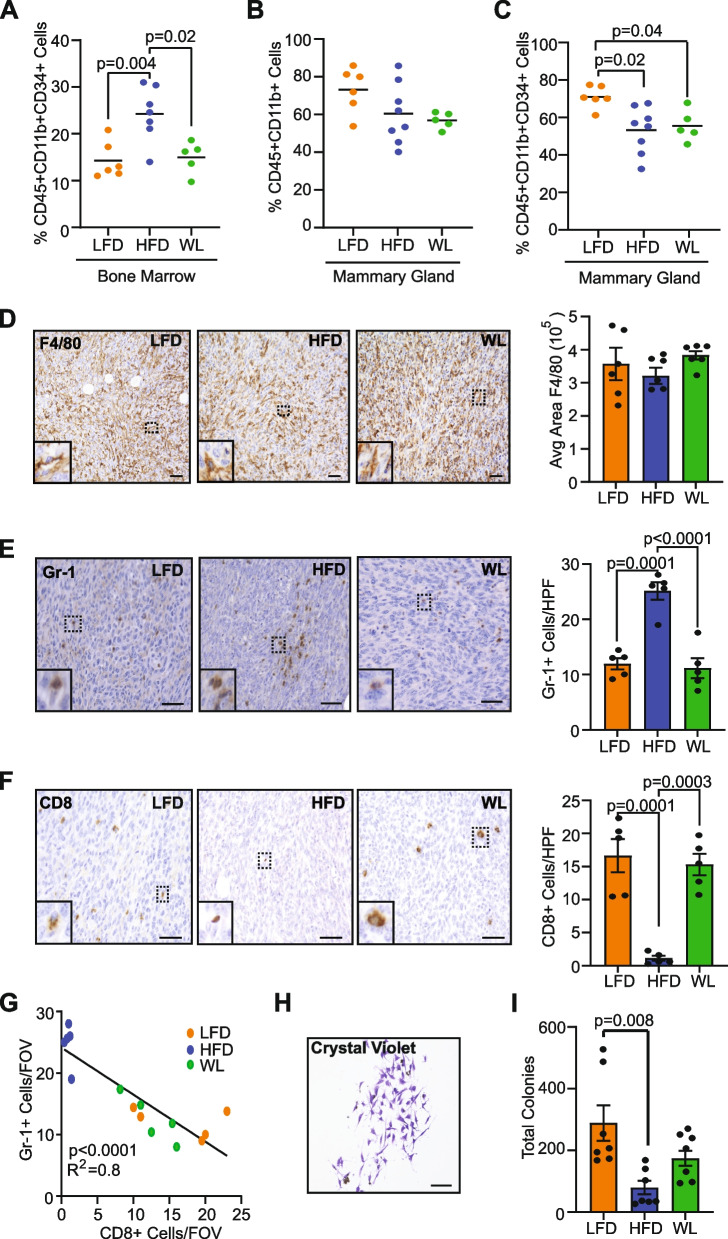
Weight loss reduces immunosuppressive microenvironment within tumors. **A** Percentage of live CD45^+^CD11b^+^CD34^+^ myeloid progenitor cells in bone marrow quantified by flow cytometry. **B** Percentage of live CD45^+^CD11b^+^ myeloid cells in TC2 tumors quantified by flow cytometry. **C** Percentage of live CD45^+^CD11b^+^CD34^+^ immature myeloid cells in TC2 tumors quantified by flow cytometry. **D** Representative images and quantification of F4/80^+^ cells in TC2 tumors (square pixels). **E** Representative images and quantification of Gr-1^+^ cells in TC2 tumors per high power field (HPF). **F** Representative images and quantification of CD8^+^ cells in TC2 tumors per HPF. **G** Correlation of Gr-1^+^ and CD8^+^ cells in tumors of LFD, HFD, and WL mice. **H** Representative crystal violet image of fibrocyte colony grown from CD11b^+^ cells isolated from TC2 tumors. **I** Quantification of total fibrocyte colonies grown from CD11b^+^ cells isolated from TC2 tumors (Kruskall-Wallis ANOVA with Dunn’s multiple comparison test). Magnification bar = 50 µm

Since we observed that obesity and weight loss impacted the myeloid progenitor cell population in the bone marrow and immature myeloid cells in the mammary gland of non-tumor-bearing mice (Fig. 2A, B), we assessed how obesity and weight loss altered these cells in tumor-bearing mice. We isolated the bone marrow from tumor-bearing lean, obese, and formerly obese mice and analyzed the immune cells using flow cytometry. Similar to non-tumor-bearing mice, obese mice had a significantly increased percentage of myeloid progenitor cells compared to lean mice (*p* = 0.004, Fig. 4A), and weight loss reduced myeloid progenitor cells compared to obese mice (*p* = 0.02, Fig. 4A). In contrast to the mammary glands of non-tumor-bearing mice, no differences were observed in the percentage of total live CD45^+^CD11b^+^ myeloid cells in mammary tumors from mice in each group (Fig. 4B). Further, the percentage of CD11b^+^CD34^+^ immature myeloid cells was decreased in tumors of both obese mice (*p* = 0.02) and weight loss mice (*p =* 0.04) compared to lean mice (Fig. 4C).

To assess myeloid lineage cells within tumors, we stained tumor sections from lean, obese, and formerly obese mice with antibodies to detect macrophages and myeloid-derived suppressor cells (MDSC). Although F4/80^+^ macrophages made up a large component of the tumor microenvironment, no significant differences were detected among the tumors from mice in each group (Fig. 4D). However, Gr-1^+^ MDSC were significantly increased in the tumors of obese mice compared to lean mice (*p* = 0.0001, Fig. 4E), and formerly obese mice had significantly reduced MDSC in tumors compared to obese mice (*p* < 0.0001, Fig. 4E). MDSC have been characterized as immunosuppressive within tumors [38, 39]. Consistent with an immunosuppressive microenvironment, CD8^+^ T cells were significantly reduced in tumors from obese mice compared to tumors from lean mice (*p* = 0.0001) or formerly obese mice (*p* = 0.0003, Fig. 4F). We also observed a significant negative correlation between Gr-1^+^ cells and CD8^+^ cells in tumors of lean, obese, and formerly obese mice (*p* < 0.0001; R^2^ = 0.8, Fig. 4G). Together, these results suggest that obesity creates an immunosuppressive tumor microenvironment, while weight loss prior to tumor growth reverses these effects.

Because we identified increased collagen deposition and CAF in tumors from obese mice, we hypothesized that obesity may also increase fibrocytes in the myeloid cell population. To test this hypothesis, we isolated CD11b^+^ cells from the tumors of lean, obese, and formerly obese mice and quantified colony formation in vitro (Fig. 4H, Additional File 4A). Interestingly, we observed significantly less total colony formation from the CD11b^+^ cells isolated from tumors (Fig. 4I) than from mammary glands (Fig. 2F). In contrast to our hypothesis, we observed significantly reduced fibrocyte colonies in CD11b^+^ cells isolated from tumors of obese mice compared to lean mice (*p* = 0.008, Fig. 4I), while fibrocyte colony formation was not significantly different among tumors of lean and formerly obese mice (Fig. 4I). These results suggest that the immature myeloid cell population in the tumor microenvironment is less enriched for fibrocytes than in the mammary glands of obese mice.

### Myeloid progenitor cells contribute to collagen deposition in mammary tumors of lean and obese mice

**Fig. 5:**
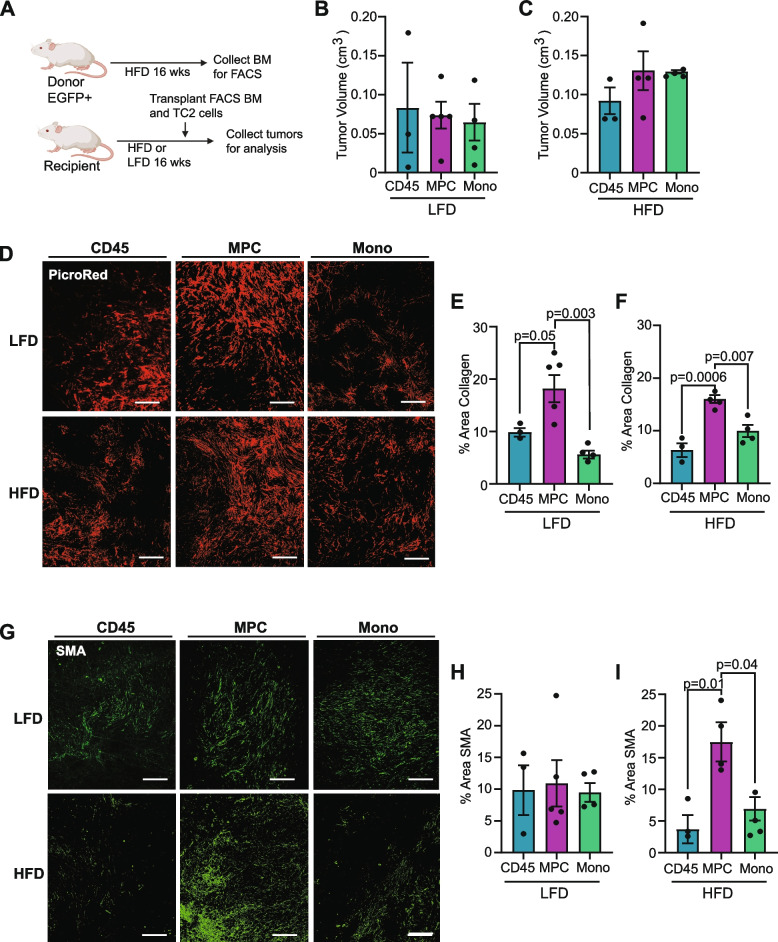
Myeloid progenitor cells contribute to TC2 mammary tumor collagen deposition, but not tumor growth. **A** Schematic of tumor transplant experiment. **B** Tumor volume of LFD-fed mice on Day 36 in groups transplanted with live CD45^+^ cells (CD45), CD11b^+^CD34^+^ myeloid progenitor cells (MPC), or CD11b^+^CD34^−^ monocytes (mono). **C** Tumor volume of HFD-fed mice on Day 25. **D** Representative images of picrosirius red-stained collagen in mammary tumors isolated from lean and obese mice. **E** Collagen within tumors normalized to total tumor area from lean mice. **F** Collagen within tumors normalized to total tumor area from obese mice. **G** Representative images of SMA expression in mammary tumors from lean and obese mice. **H** SMA expression within tumors normalized to total tumor area from lean mice. **I** SMA expression within tumors normalized to total tumor area from obese mice. Magnification bar: (**D, G**) 300 µm

We have previously observed that immature myeloid cells isolated from the mammary glands of obese mice expressed collagen 1 and collagen 3 [17], which are believed to be defining features of fibrocytes that are absent from other hematopoietic cells [40]. Since we observed increased collagen deposition and CAF within the tumors of obese mice, we hypothesized that fibrocytes present within the mammary glands during tumor formation may promote a more fibrotic tumor microenvironment. To test this hypothesis, EGFP^+^ FVB/N mice, which ubiquitously express enhanced GFP [27], were fed HFD for 16 weeks to induce obesity. Using FACS, we then isolated total CD45^+^ cells (CD45), CD11b^+^CD34^−^ monocytes (Mono), and CD11b^+^CD34^+^ myeloid progenitor cells (MPC), which are the cells of origin of fibrocytes, from the bone marrow of obese EGFP^+^ donor mice. TC2 mammary tumor cells were mixed with the sorted bone marrow populations, then injected into the mammary glands of lean and obese mice (Fig. 5A). Tumors were collected when the largest tumor in each group reached 0.7 cm in diameter. Tumors from LFD-fed mice grew significantly slower and were collected after 36 days compared to tumors from HFD-fed mice, which were collected after 25 days. When bone marrow populations were transplanted with tumor cells, tumor growth was not significantly different in any group in lean or obese mice (Fig. 5B, C).

To assess the impact of the isolated bone marrow cell populations on the mammary tumor microenvironment, collagen deposition was quantified within tumors from each group using picrosirius red staining (Fig. 5D). Transplant of myeloid progenitor cells with the tumor cells significantly increased collagen within tumors from both lean (*p* = 0.003, Fig. 5E) and obese (*p* = 0.007, Fig. 5F) mice, compared to the more differentiated CD11b^+^CD34^−^ population of monocytes. To quantify CAF, tumors from all groups were stained for SMA (Fig. 5G). While no significant differences were observed in tumors from lean mice (Fig. 5H), SMA was significantly increased in tumors grown with myeloid progenitor cells from obese mice, compared to those transplanted with total CD45^+^ cells (*p* = 0.01) or CD11b^+^CD34^−^ monocytes (*p* = 0.04, Fig. 5I). Together, these results suggest that the increased fibrocytes present at the time of tumor formation promote the development of desmoplastic stroma in obese mice.

Fibrocytes have been shown to differentiate into myofibroblasts in mouse models of other types of cancer [41, 42] or stimulate other cells to promote fibrosis [43, 44]. To identify whether the transplanted EGFP^+^ cells contributed to the CAF population, we dissociated tumors and quantified GFP expression using flow cytometry. We did not detect a significant number of GFP^+^ cells in the tumors from any group of mice (Additional File 4B).

## Discussion

Obesity is associated with poor breast cancer prognosis [6]. While weight loss improves outcomes for other health conditions, little is known about how weight loss prior to tumor formation potentially impacts the growth and microenvironment of mammary tumors. Our studies suggest that while weight loss did not completely reduce the rate of tumor growth, the microenvironment of the resulting tumors was less fibrotic and immunosuppressive than tumors from obese mice. While fibrocytes appear to be less frequent in growing tumors, their presence in the mammary gland prior to tumor formation may promote the rapid formation of CAF in the early tumor microenvironment. We observed that mixing of myeloid progenitor cells from the bone marrow of obese mice with TC2 tumors cells resulted in significantly increased collagen deposition in tumors of both lean and obese mice. Fibrocytes may promote fibrotic changes in resident fibroblasts and adipose-derived stromal cells to become CAF in the developing tumor microenvironment, leading to more desmoplastic tumors observed clinically in breast cancer patients with obesity [7].

Within the mammary glands of non-tumor-bearing mice, weight loss did not resolve the increased collagen deposition around mammary ducts, indicating that fibrosis may be a longer-lasting microenvironmental condition than inflammation due to macrophages in CLS. These results are consistent with human studies of fibrosis in subcutaneous and visceral fat following weight loss through bariatric surgery [14, 15]. The continued presence of elevated collagen in the mammary glands of formerly obese mice may reflect slower tissue remodeling of mature collagen fibers within the mammary gland [45].

Consistent with a decrease in myeloid progenitor cells in the bone marrow, we observed a decrease in fibrocytes within the mammary glands of formerly obese mice. Multiple signals have been shown to enhance fibrocyte recruitment into fibrotic conditions including chemokine (C-X-C motif) ligand 12 (CXCL12) and platelet-derived growth factor receptor [46–49]. Adipose tissue expression of CCL2 is increased in obesity [50], and we have shown that loss of CCR2 signaling reduces fibrocytes within the obese mammary gland [17]. Weight loss has been shown to decrease circulating levels of CCL2 [51], which may lead to the decreased recruitment of fibrocytes that we observed in the mammary glands of formerly obese mice. However, little is known about how fibrocyte numbers are regulated in the myeloid progenitor cell population within the bone marrow. Inflammatory cytokines, including interleukin (IL)-6, tumor necrosis factor alpha (TNFα), and IL-1β, have been shown promote the expansion of myeloid progenitor cells [52, 53] and are produced by both adipocytes and macrophages in obesity [54–56]. Direct effects of these cytokines on fibrocytes or fibrocyte progenitor cells has not been examined.

While we observed that weight loss reduced CLS, which are a histological marker for local inflammation [57], the total CD11b^+^ cell population, which includes macrophages, was not reduced. Studies of macrophages within different adipose tissue depots have demonstrated that the macrophage population is heterogeneous, depending on microenvironment conditions [58–60]. Macrophages that form CLS may be functionally distinct from macrophages in other locations within adipose tissue [61]. Macrophages can also acquire a metabolically-activated phenotype [62] due to removal of lipid from dying adipocytes [63]. In visceral fat during weight loss, macrophage populations shift to include those with a phagocytotic function, which may participate in tissue remodeling [59, 64]. Further, in the obesity-resistant Balb/c strain, mice that switched from a HFD to a LFD had F4/80^+^ macrophages that remained elevated in the mammary glands [65], which may suggest that exposure to a HFD may also play a role in the macrophage populations present. The estrus cycle also contributes to immune cells regulation in the mammary gland [66]. Since we did not examine immune cell populations at a synchronized point in the estrus cycle, this limitation may have contributed to variability we observed in our study. CD11b is expressed at various levels by multiple different cell types [67], and additional gene expression experiments and expanded flow cytometry markers are required to characterize the distinct populations of CD11b^+^ cells and their localization in the mammary glands of lean, obese, and formerly obese mice.

Similar to the CD11b^+^ cell population in the mammary gland, the CD11b^+^ cell population in mammary tumors is heterogeneous. The growing mammary tumor may affect both the types and functions of myeloid cells within the tumor microenvironment [68]. Further, through cytokine secretion, mammary tumors can increase proliferation of specific populations of myeloid cells within bone marrow [69, 70], which may change the composition of myeloid cells recruited into tumors. Although we observed an increase in fibrocytes within the CD11b^+^ cell population in the mammary glands of obese non-tumor-bearing mice, fibrocytes were no longer enhanced within this population in the tumor microenvironment. Cytokines and growth factors secreted by tumor cells may shift the differentiation of immature myeloid cells into MDSC and tumor-associated macrophages [71]. Consistent with this idea, we observed increased Gr-1^+^ MDSC in the tumors of obese mice, and the immunosuppressive environment was reflected in significantly reduced CD8^+^ T cells. Granulocyte–macrophage colony-stimulating factor is one cytokine that has been implicated in promoting the differentiation of MDSC from immature myeloid cells within tumors [72, 73]. While MDSC are elevated in tumors, it is likely that multiple factors contribute to an immunosuppressive microenvironment. Following treatment with anti-F4/80 antibodies to deplete macrophages, both obese and lean mice had elevated levels of CD8^+^ T cells within tumors [36]. Further work is necessary to identify how obesity alters the recruitment and function of immune cells within tumors contributing to immunosuppression.

Recent work suggests that fibrocytes play an important role in lung tumor growth and generation of metastases [22]. When transplanted with gastric cancer cells, fibrocytes promoted the growth of larger, more fibrotic tumors [42]. Following transplantation of myeloid progenitor cells with TC2 tumor cells, we did not observe significant promotion of tumor growth compared to the other groups. However, collagen deposition was significantly increased in the tumors transplanted with myeloid progenitor cells in both obese and lean mice. While we hypothesized that fibrocytes differentiated into myofibroblasts within the tumors, we did not detect GFP^+^ cells in tumors from any group. These data could suggest that fibrocytes had an impact in tumors early during growth and recruited limited fibrocytes from circulation as the tumors grew. Interestingly, we observed a significant increase in SMA^+^ CAF in obese, but not lean mice. Given the number of cytokines associated with fibrocyte recruitment and differentiation that are upregulated in obesity [46–49, 74–76], fibrocytes from CD11b^+^CD34^+^ cells could be longer lived in obese mice, leading to increased SMA^+^ CAFs in tumors of obese mice. Transplanted fibrocytes may also induce the differentiation or recruitment of other cells to become CAFs, as has been observed in culture in other contexts [43, 44]. It is also possible that this experiment was limited by technical challenges such as rejection of GFP^+^ immune cells by recipient mice. Further insight into the role of fibrocytes in mammary tumor growth may be gained through lineage tracing studies as well examining tumors at earlier time points during tumor growth.

Weight loss is a commonly recommended intervention for obesity and may reduce breast cancer risk [8, 9]. Multiple methods for weight loss are clinically used, and each method for weight loss may impact the mammary gland and the resulting tumor microenvironment in divergent ways. In a recent study that explored four different methods for weight loss, mice that lost weight through low-fat calorie restriction, Mediterranean-style calorie restriction, and intermittent-calorie restriction had reduced tumor growth and diminished expression of genes associated with epithelial-to-mesenchymal transition following injection with EO771 mammary tumor cells compared to mice fed a HFD or formerly obese mice that lost weight through switching to a non-restricted LFD [77]. In a mouse model using bariatric surgery to induce weight loss, mammary tumors that developed formerly obese mice in the surgical group had higher expression of genes associated with an inflammatory response and improved responses to anti-PD-L1 immune checkpoint therapy [78]. Together these studies suggest that the method for weight loss may have a long-term impact on the biology of tumors that may develop at a later time point. It is also possible that changes in the mammary microenvironment due to weight loss may have divergent effects on different subtypes of breast cancer. In models of triple negative breast cancer, mice that lost weight due to decreased consumption of a HFD developed tumors with aggressive characteristics that were more similar to tumors from obese mice [77, 79]. Consistent with our study, weight loss did not decrease the latency to tumor formation compared to obese mice [77, 79]. Here we show that weight loss prior to ERα^+^ tumor formation limits desmoplasia and immunosuppression within mammary tumors. Since increased desmoplasia, including SMA^+^ stromal cells, is associated with worse survival in breast cancer patients [80, 81], our results suggest that weight loss prior to ERα^+^ mammary tumor formation may ameliorate the effects of obesity on the tumor microenvironment. Understanding how different methods of weight loss alter the microenvironment and biology of tumors may lead to improved prevention strategies for women at high risk for breast cancer.

## Conclusions

Obesity is a risk factor for breast cancer in postmenopausal women. Regardless of menopausal status, women with obesity diagnosed with breast cancer have a worsened prognosis. Here, we show that weight loss reduces the formation of inflammatory CLS but does not decrease myeloid cells within the mammary gland. While the total collagen surrounding the mammary ducts is not decreased with weight loss, fibrocytes show diminished recruitment into the mammary glands of formerly obese mice compared to obese mice. These results suggest that collagen deposition could decrease following a longer period of time where weight loss is sustained. We also observed that the microenvironment of the tumors that formed in formerly obese mice were more similar to tumors from lean mice than obese mice, suggestive of the growth of less aggressive tumors. Further work is necessary to identify how different methods of weight loss impact the mammary tumor microenvironment as well as how weight loss alters the formation of tumors of distinct subtypes.

### Supplementary Information

**Additional file 1.** Antibodies used for flow cytometry, immunofluorescence, and immunohistochemistry.

**Additional file 2.** Identification of macrophages and collagen in the mammary gland. (A) Representative images of F4/80-stained mammary glands from low-fat diet (LFD), high-fat diet (HFD), and weight loss (WL) groups. (B) Representative images of CD11b-stained mammary glands from LFD, HFD, and WL groups. (C) Flow cytometry gating strategy of mammary gland. Cells were gated for debris, followed by single cells, and live cells using viability dye. Live cells were gated for CD45 and CD11b expression, and CD11b^+^ cells were further gated for CD34 expression. (D) Representative images of picrosirius red stained mammary glands from LFD, HFD, and WL groups. (E) Duct area measured in square pixels. (F) Collagen area measured in square pixels. Magnification bar=100 µm.

**Additional file 3.** Identification of fibrocytes. (A) Flow cytometry gating strategy of bone marrow. Cells were gated for debris, followed by single cells, and live cells using viability dye. Live cells were gated for CD45 and CD11b expression, and CD11b^+^ cells were further gated for CD34 expression. (B) Representative image of cells in fibrocyte colony stained with F4/80, alpha-smooth muscle actin (SMA) and DAPI. (C) Representative image of cells in fibrocyte colony stained with CD45 and DAPI. (D) Representative images of SMA, CD11b, and DAPI staining in mammary glands of LFD, HFD, and WL groups. Magnification bar: (B) 25 µm; (C) 50 µm; (D) 100 µm.

**Additional file 4.** Fibrocytes in tumors. (A) Representative images of fibrocyte colonies from isolated CD11b^+^ cells from tumors from LFD, HFD, and WL groups. (B) Flow cytometry gating strategy to detect GFP in TC2 tumors mixed with bone marrow cell populations. Cells were gated for debris, followed by single cells, and then gated for GFP expression. Magnification bar=100 µm.

## Data Availability

All data generated or analyzed during this study are included in this published article.

## Abbreviations

CAF: Cancer-associated fibroblasts
CLS: Crown-like structures
DCIS: Ductal carcinoma in situ
EGFP: Enhanced green fluorescent protein
ERα: Estrogen receptor alpha
HFD: High-fat diet
LFD: Low-fat diet
MDSC: Myeloid-derived suppressor cells
SMA: Alpha smooth muscle actin
