# Supplementary material for: Alterations in the mammary gland and tumor microenvironment of formerly obese mice
Source: BMC Cancer. 2023 Dec 1;23:1183. doi: 10.1186/s12885-023-11688-3 (PMC10693119; doi:10.1186/s12885-023-11688-3)
Supplement: Supplementary file 1 — Additional file 1. Antibodies used for flow cytometry, immunofluorescence, and immunohistochemistry. [file 12885_2023_11688_MOESM1_ESM.docx]

**Additional File 1.** Antibodies used for flow cytometry, immunofluorescence, and immunohistochemistry.

|  | **Concentration** | **Catalog Number** | **Supplier** | **RRID** |
| --- | --- | --- | --- | --- |
| Fixable Viability Dye eFluor780 | 1:1000 | 65-0865 | ThermoFisher |  |
| CD16/32 | 0.5 ng/µl | 14-0161-85 | ThermoFisher | AB_467133 |
| BV421 CD34 | 16 ng/µl | 562608 | BD Biosciences | AB_11154576 |
| APC CD11b | 2.5 ng/µl | 17-0112-82 | ThermoFisher | AB_469343 |
| PE CD45 | 2.5 ng/µl | 12-0451-82 | ThermoFisher | AB_465668 |
| CD11b | 0.1 µg/µl | 14-0112-85 | ThermoFisher | AB_467108 |
| F4/80 | 1:250 | 123102 | BioLegend | AB_893506 |
| CD11b | 1:200 | NB110-89474 | Novus | AB_1216361 |
| CD45 | 1:250 | 103101 | BioLegend | AB_312966 |
| α-Smooth Muscle Actin (SMA) | 1:1500 | A5228 | Sigma-Aldrich | AB_262054 |
| Collagen I | 1:200 | NB600-408 | Novus | AB_10000511 |
| Estrogen Receptor α | 1:500 | 06-935 | EMD Millipore | AB_310305 |
| Platelet-derived growth factor receptor alpha (PDGFRα) | 1:200 | PA5-16742 | ThermoFisher | AB_10980345 |
| Ly-6G / Ly-6C | 1:200 | MA1-10401 | ThermoFisher | AB_11152791 |
| CD8 | 1:200 | NBP1-49045 | Novus | AB_10011327 |
| 488 anti-Mouse | 1:300 | A11001 | ThermoFisher | AB_2534069 |
| 488 anti-Goat | 1:300 | A11055 | ThermoFisher | AB_2534102 |
| 546 anti-Mouse | 1:300 | A11003 | ThermoFisher | AB_2534071 |
| 546 anti-Rabbit | 1:300 | A11010 | ThermoFisher | AB_2534077 |
| Biotinylated anti-Rabbit | 1:500 | BA-1000 | Vector | AB_2313606 |
| Biotinylated anti-Rat | 1:250 | BA-4000 | Vector | AB_2336206 |
