# Supplementary figures and images for: Alterations in the mammary gland and tumor microenvironment of formerly obese mice
Source: BMC Cancer. 2023 Dec 1;23:1183. doi: 10.1186/s12885-023-11688-3 (PMC10693119; doi:10.1186/s12885-023-11688-3)

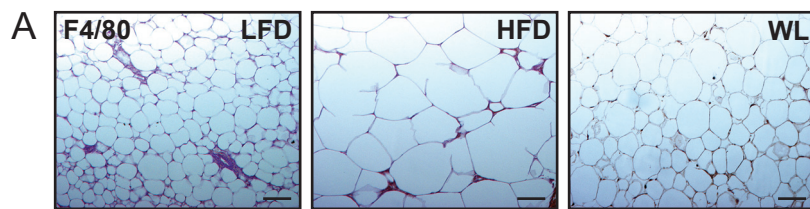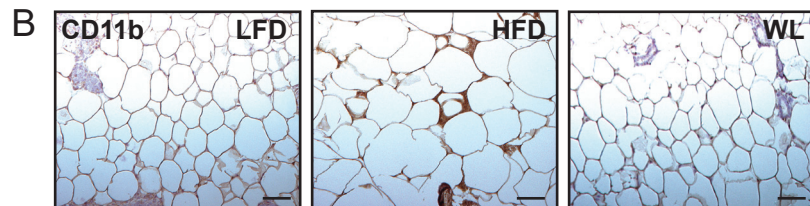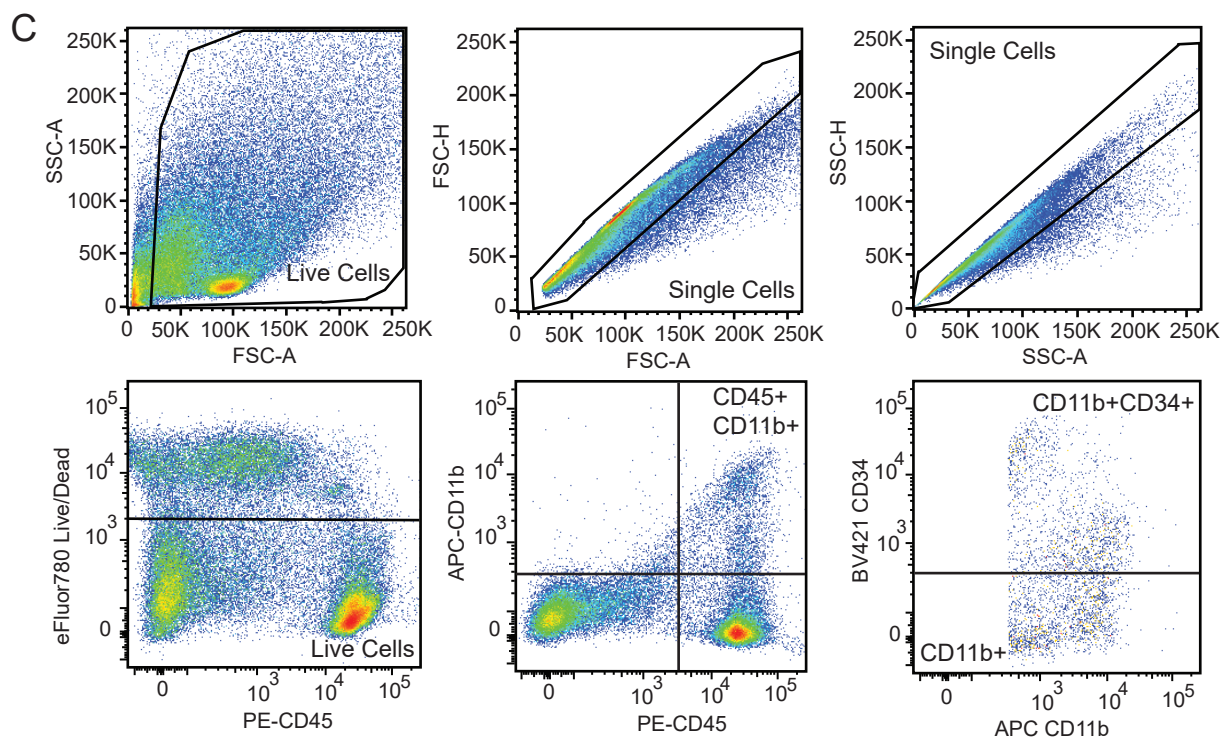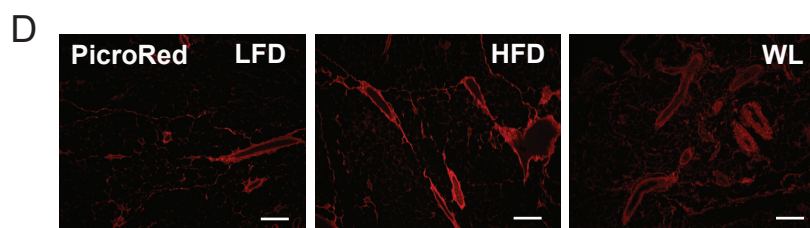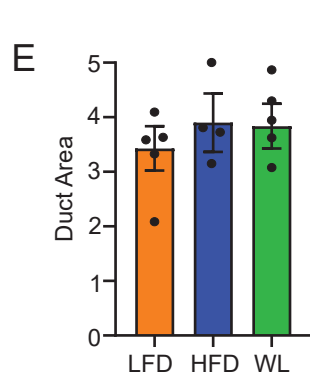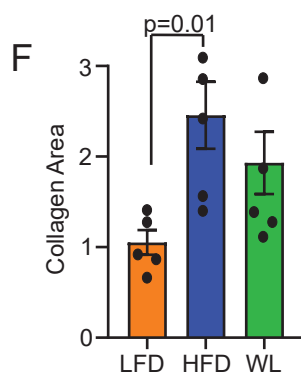

Supplement: Supplementary file 2 — Additional file 2. Identification of macrophages and collagen in the mammary gland. (A) Representative images of F4/80-stained mammary glands from low-fat diet (LFD), high-fat diet (HFD), and weight loss (WL) groups. (B) Representative images of CD11b-stained mammary glands from LFD, HFD, and WL groups. (C) Flow cytometry gating strategy of mammary gland. Cells were gated for debris, followed by single cells, and live cells using viability dye. Live cells were gated for CD45 and CD11b expression, and CD11b+ cells were further gated for CD34 expression. (D) Representative images of picrosirius red stained mammary glands from LFD, HFD, and WL groups. (E) Duct area measured in square pixels. (F) Collagen area measured in square pixels. Magnification bar=100 µm. [file 12885_2023_11688_MOESM2_ESM.pdf]

**A**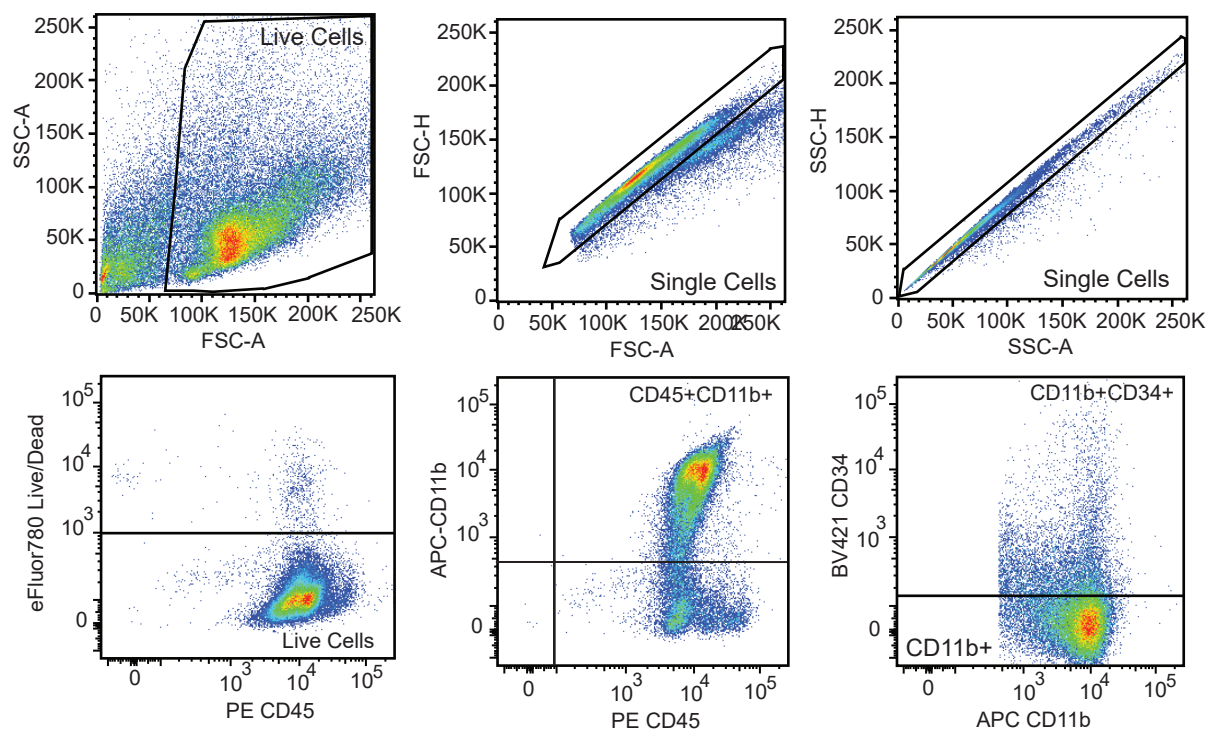**B**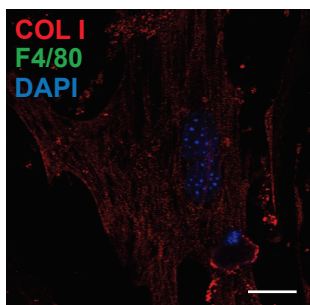**C**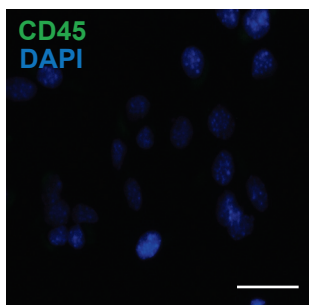**D**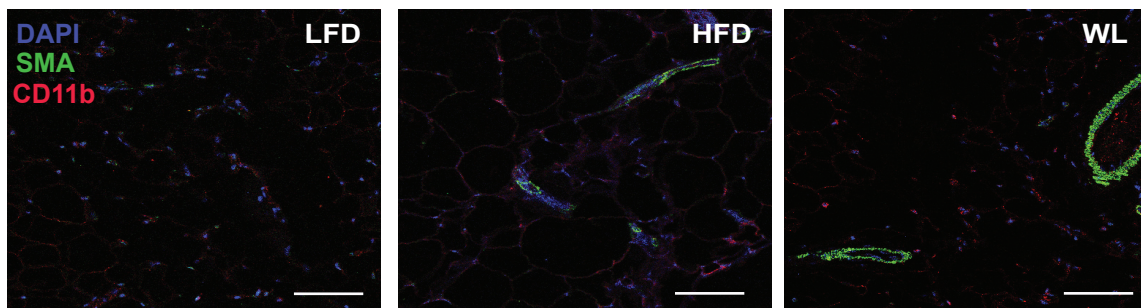

Supplement: Supplementary file 3 — Additional file 3. Identification of fibrocytes. (A) Flow cytometry gating strategy of bone marrow. Cells were gated for debris, followed by single cells, and live cells using viability dye. Live cells were gated for CD45 and CD11b expression, and CD11b+ cells were further gated for CD34 expression. (B) Representative image of cells in fibrocyte colony stained with F4/80, alpha-smooth muscle actin (SMA) and DAPI. (C) Representative image of cells in fibrocyte colony stained with CD45 and DAPI. (D) Representative images of SMA, CD11b, and DAPI staining in mammary glands of LFD, HFD, and WL groups. Magnification bar: (B) 25 µm; (C) 50 µm; (D) 100 µm. [file 12885_2023_11688_MOESM3_ESM.pdf]

A

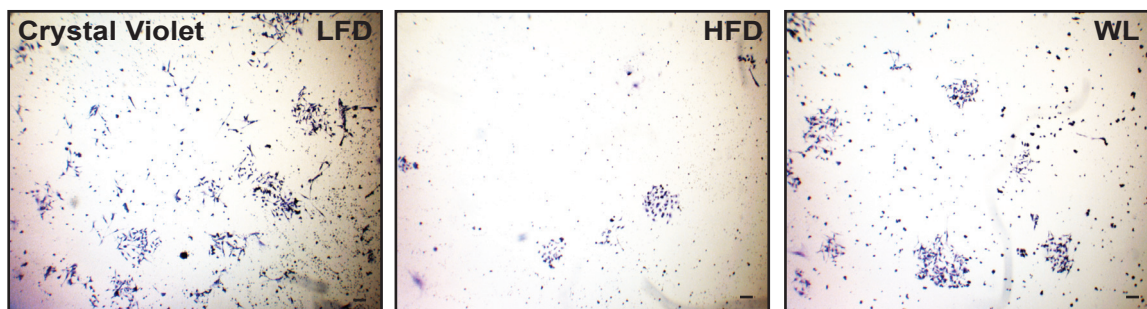

B

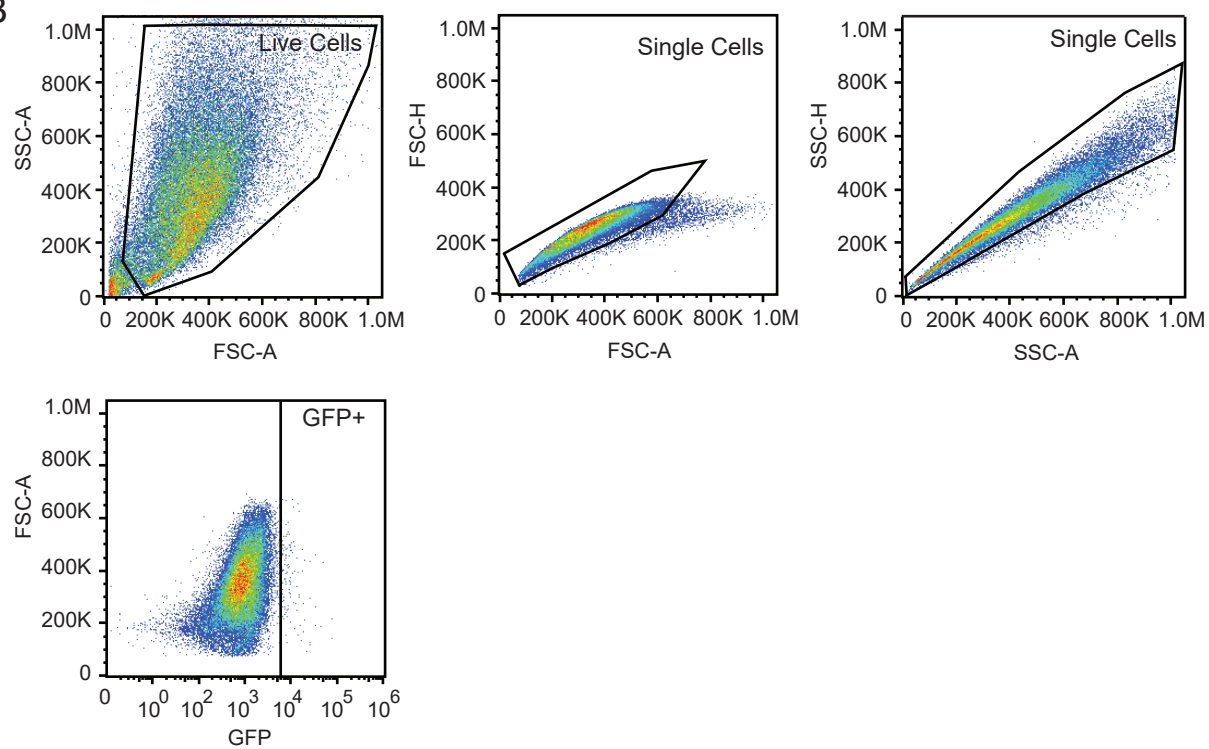

Supplement: Supplementary file 4 — Additional file 4. Fibrocytes in tumors. (A) Representative images of fibrocyte colonies from isolated CD11b+ cells from tumors from LFD, HFD, and WL groups. (B) Flow cytometry gating strategy to detect GFP in TC2 tumors mixed with bone marrow cell populations. Cells were gated for debris, followed by single cells, and then gated for GFP expression. Magnification bar=100 µm. [file 12885_2023_11688_MOESM4_ESM.pdf]
